# Plague: Past, Present, and Future

**DOI:** 10.1371/journal.pmed.0050003

**Published:** 2008-01-15

**Authors:** Nils Chr Stenseth, Bakyt B Atshabar, Mike Begon, Steven R Belmain, Eric Bertherat, Elisabeth Carniel, Kenneth L Gage, Herwig Leirs, Lila Rahalison

## Abstract

The authors argue that plague should be taken much more seriously by the international health community.

Recent experience with SARS (severe acute respiratory syndrome) [1] and avian flu shows that the public and political response to threats from new anthropozoonoses can be near-hysteria. This can readily make us forget more classical animal-borne diseases, such as plague (Box 1).

**Box 1. The Plague**

The causative bacterium (Yersinia pestis) was discovered by Yersin in 1894 [11] (see also [63]). Case-fatality ratio varies from 30% to 100%, if left untreated. Plague is endemic in many countries in the Americas, Asia, and Africa. More than 90% of cases are currently being reported from Africa.

**Clinical presentation:** After an incubation period of 3–7 days, patients typically experience a sudden onset of fever, chills, headaches, body aches, weakness, vomiting, and nausea. Clinical plague infection manifests itself in three forms, depending on the route of infection: bubonic, septicaemic, and pneumonic. The bubonic form is the most common, resulting from the bite of an infected flea. The pneumonic form initially is directly transmitted from human to human via inhalation of infected respiratory droplets.

**Treatment:** Rapid diagnosis and treatment are essential to reduce the risk of complications and death. Streptomycin, tetracyclines, and sulfonamides are the standard treatment. Gentamicin and fluoroquinolones may represent alternatives when the above antibiotics are not available. Patients with pneumonic plague must be isolated to avoid respiratory transmission.

**Challenges ahead:** Biological diagnosis of plague remains a challenge because most human cases appear in remote areas with scarce laboratory resources. So far, the main confirmation techniques were based on the isolation of Y. pestis (requiring a minimum of 4 days). The recent development of rapid diagnostic tests, now considered a confirmation method in endemic areas, opens new possibilities in terms of surveillance and case management.

Three recent international meetings on plague (Box 2) concluded that: (1) it should be re-emphasised that the plague bacillus (Yersinia pestis) still causes several thousand human cases per year [2,3] (Figure 1); (2) locally perceived risks far outstrip the objective risk based purely on the number of cases [2]; (3) climate change might increase the risk of plague outbreaks where plague is currently endemic and new plague areas might arise [2,4]; (4) remarkably little is known about the dynamics of plague in its natural reservoirs and hence about changing risks for humans [5]; and, therefore, (5) plague should be taken much more seriously by the international community than appears to be the case.

**Figure 1:**
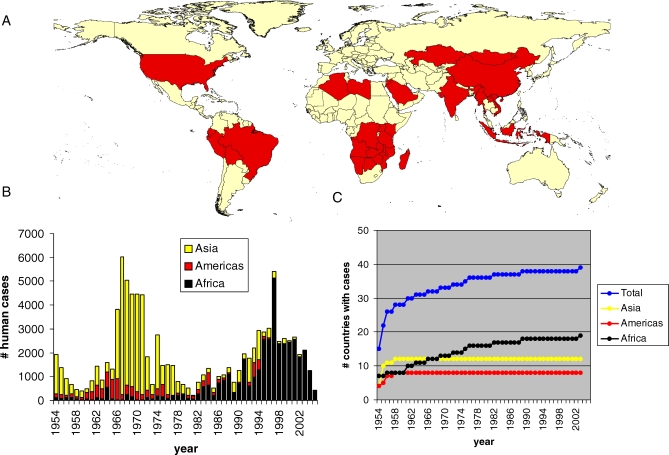
The Global Distribution of Plague (A) Map showing countries with known presence of plague in wild reservoir species (red) (after [3]). For US only the mainland below 50° N is shown. (B) Annual number of human plague cases over different continents, reported to WHO in the period 1954–2005. (C) Cumulative number of countries that reported plague to WHO since 1954.

**Box 2. Recent International Meetings on Plague**

- A meeting on plague in the present, past, and future was held by the Academy of Science and Letters, Oslo, Norway (http://www.cees.no/oslo-plague-meeting).
- A workshop focusing on the comparison of the Black Death and modern plague was organised by the Wellcome Trust Centre for the History of Medicine, University College, London (http://www.ucl.ac.uk/histmed/).
- The World Health Organization convened an expert meeting in Antananarivo, Madagascar updating clinical plague definitions, diagnostic methods, vaccines, and antimicrobial therapy, as well as reservoir and vector control strategies (http://www.who.int/csr/disease/plague/interregionalmeeting2006/en/index.html).

## The Plague Eco-Epidemiological System

The plague bacillus causes a rapidly progressing, serious illness that in its bubonic form is likely to be fatal (40%–70% mortality). Without prompt antibiotic treatment, pneumonic and septicaemic plague are virtually always fatal. For these reasons Y. pestis is considered one of the most pathogenic bacteria for humans.

Yersinia pestis is transmitted by fleas, while the other two species of Yersinia known to be pathogenic for humans (Y. enterocolitica and Y. pseudotuberculosis) are transmitted by the faecal–oral route and cause intestinal symptoms of moderate intensity. Yersinia pestis is believed to be a clone of Y. pseudotuberculosis that emerged within the last 1,500 to 20,000 years [6,7]. This divergence was characterised by the acquisition of a few genetic elements; more particularly, two plasmids that play a key role in flea-borne transmission [8,9]. The exceptional pathogenicity of Y. pestis compared to the enteropathogenic species may be explained by its new mode of transmission. Indeed, the only means for this bacterium to be transferred to new hosts is through septicaemia, which allows the bacteria present in the bloodstream to be efficiently taken up by the flea during its blood meal [10].

Soon after Yersin's identification of the plague bacillus [11], it became clear that urban outbreaks were linked to transmission among commensal rats and their fleas. In this classic urban-plague scenario, infected rats (transported, for example, by ships) arrive in a new city and transmit the infection to local house rats and their fleas, which then serve as sources of human infection. Occasionally, humans develop a pneumonic form of plague that is then directly transmitted from human to human through respiratory droplets.

**Figure 2:**
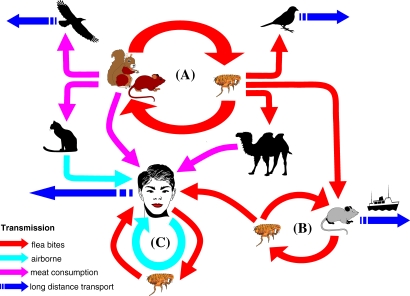
Possible Transmission Pathways for the Plague Agent, Y. pestis These pathways include sylvatic rodent-flea cycles (A), the commensal rodent-flea cycles (B), and the pneumonic transmission in humans (C). The colour of the arrows indicates the mechanism (flea bites, air particles, meat consumption) through which the bacteria are transferred from one host to another. Dark blue arrows indicate ways in which plague can move to other areas.

The epidemiology of plague, however, is much more complicated than this urban-plague scenario suggests, involving several other—more likely—pathways of transmission (Box 3 and Figure 2). This complicated epidemiology necessitates a reconsideration of plague ecology.

**Box 3. The Plague Bacterium within the Ecological Web**

Maintenance of plague foci depends on a whole suite of rodent hosts and their associated fleas (Figure 2A). Under favourable conditions, the plague bacillus might survive in the environment, essentially in rodent burrows [64]. When an infected flea happens to feed on a commensal rodent, the cycle continues in the latter (Figure 2B). As commensal rodents die, their fleas are forced to move to alternative hosts, e.g., humans. If humans develop pneumonic plague, the infection may be transmitted from person to person through respiratory droplets spread by coughing (Figure 2C). Humans may also become infected through handling of infected animals (or meat), including rodents, camels, or cats. Cats can also develop pneumonic plague, passing their infection to their owners through coughing. There is, finally, some evidence suggesting that the human flea, Pulex irritans, can be involved in human-to-human transmission [37]. Mammal predators, birds of prey, and other birds that use rodent burrows for nesting may move over larger areas than the rodents themselves, spreading the infection over longer distances. Also, infected commensal rats or humans may travel over long distances.

## The Past

Plague has given rise to at least three major pandemics. The first (“the Justinian plague”) spread around the Mediterranean Sea in the 6th century AD, the second (“the Black Death”) started in Europe in the 14th century and recurred intermittently for more than 300 years, and the third started in China during the middle of the 19th century and spread throughout the world. Purportedly, each pandemic was caused by a different biovar of Y. pestis, respectively, Antiqua (still found in Africa and Central Asia), Medievalis (currently limited to Central Asia), and Orientalis (almost worldwide in its distribution) [12,13].

The Black Death decimated Medieval Europe, and had a major impact on the continent's socio-economic development, culture, art, religion, and politics [14,15]. Several high-profile critiques have questioned whether the Black Death was indeed caused by Y. pestis [16,17]. Proponents of both sides of the debate came together at last year's Oslo and London meetings (Box 2). It was generally accepted that the epidemiology of Black Death plague, as reflected in historical records, did not always match the “classical” rat-flea-human plague cycle. However, the reported medical symptoms were very similar during each pandemic. In addition, the international experience of modern-day plague represented at the Oslo meeting made it clear that “classical” plague epidemiology is only one of several possibilities to explain the Black Death, and may not even be the most typical of the different plague ecology systems [18]. Discovery of Y. pestis genetic material in those who died from the Black Death and are buried in medieval graves [19] further supports the view that Y. pestis was the causative agent of the Black Death.

## The Present

Given this history, plague is often classified as a problem of the past. However, it remains a current threat in many parts of the world (Figure 1A), particularly in Africa, where both the number of cases (Figure 1B) and the number of countries reporting plague (Figure 1C) have increased during recent decades. Following the reappearance of plague during the 1990s in several countries, plague has been categorised as a re-emerging disease [20,21].

Plague is endemic in a variety of wildlife rodent species worldwide in a wide range of natural habitats, with commensal rats only sometimes playing a role as “liaison” hosts, carrying plague between the sylvatic reservoir and people. Various other animal-to-human transmission pathways have been documented. Human plague may be contracted from (1) being bitten by the fleas of wildlife rodent species in rural settings (e.g., in the south-western United States [22,23]) or of commensal rodents that move freely between villages and the forest habitats occupied by reservoir hosts (e.g., in Tanzania); rodents' movements have become more frequent as human activity has fragmented the forest [24]; (2) eating infected animals such as guinea pigs in Peru and Ecuador [25,26] or camels that contract the disease from rodent fleas in Central Asia and the Middle East [27–31]; or (3) handling cats infected through the consumption of plague-infected rodents in Africa or the United States [32–34]. Human-to-human transmission also occurs, either directly through respiratory droplets or indirectly via flea bites [35–37].

Over the last 20 years, there have been 1,000 to 5,000 human cases of plague and 100 to 200 deaths reported to the World Health Organization (WHO) each year [38]. However, because of poor diagnostic facilities and underreporting, the number of cases is almost certainly much higher. Over the years, there has been a major shift in cases from Asia to Africa (Figure 1B), with more than 90% of all cases and deaths in the last five years occurring in Madagascar, Tanzania, Mozambique, Malawi, Uganda, and the Democratic Republic of the Congo (DRC). Most are cases of bubonic plague contracted through contact with infected rodents and fleas. However, outbreaks of pneumonic plague still occur: the most recent large one was in October and November 2006 in DRC, with hundreds of suspected cases [39], and a smaller outbreak arose just across the border in nearby Uganda in February 2007 ([40]). In December 2004 there was a pneumonic outbreak in a miners' camp in DRC, probably imported by an infected human who had travelled from an endemic zone. One pneumonic case even arrived in Kisangani, a large city several hundred kilometres away [41,42]. So even rapid-reaction medical teams may not be sufficient to stop plague from spreading quickly over long distances before it is detected and managed.

Africa is particularly at risk for a number of reasons. Poor rural communities typically live in close proximity to rodents, which are widely hunted and eaten in many plague-endemic areas. Superstitions, lack of money, and distance from health facilities often lead to delays in seeking health care and receiving treatment. The public health system in large parts of Africa is poorly organised and equipped, and political crisis and social disorganisation impede improvements. Finally, anthropogenic changes to the landscape and to patterns of human mobility are increasingly favouring contact between plague-reservoir and peridomestic rodents, and between people from plague-endemic and previously unaffected regions.

## Looking to the Future

Plague cannot be eradicated, since it is widespread in wildlife rodent reservoirs. Hence, there is a critical need to understand how human risks are affected by the dynamics of these wildlife reservoirs. For example, the likelihood of a plague outbreak in North American and Central Asian rodents, and the resulting risk to humans, is known to be affected by climate [43,44]. Recent analysis of data from Kazakhstan [45] shows that warmer springs and wetter summers increase the prevalence of plague in its main host, the great gerbil. Such environmental conditions also seem to have prevailed during the emergence of the Second and Third Pandemics [46,47]—conditions that might become more common in the future [48].

Although the number of human cases of plague is relatively low, it would be a mistake to overlook its threat to humanity, because of the disease's inherent communicability, rapid spread, rapid clinical course, and high mortality if left untreated. A plague outbreak may also cause widespread panic, as occurred in India in 1994 when a relatively small outbreak, with 50 deaths, was reported in the city of Surat [49]. This led to a nationwide collapse in tourism and trade, with an estimated cost of US$600 million [50].

Outbreaks are usually tackled with a fire-fighting approach. Teams move into an infected area to kill fleas with insecticides, treat human cases, and give chemoprophylaxis to exposed people. Many experts have argued that this crisis-management approach is insufficient as the outbreak is likely to be on the wane by the time action is taken. Informed, pre-emptive decisions about plague management and prevention before outbreaks occur would certainly be more sustainable and cost-beneficial. There has been some recent progress, such as development of rapid diagnosis tools [51], some challenging of accepted dogma about the dynamics of sylvatic plague in the United States [52] and in Central Asia [53], and the identification of predictive critical rodent abundance thresholds for plague in Kazakhstan [54]. What is striking, though, is our lack of understanding of this high-profile disease in even the best-studied foci, particularly in Africa: often, we do not even know the natural reservoir of the bacilli.

The capacity of the plague bacillus to form permanent foci under highly diverse ecological conditions attests to its extraordinary adaptability. During its emergence in Central Asia, Y. pestis accumulated copies of insertion sequences rendering its genome highly plastic [55]. The capacity to undergo genomic rearrangements may thus be an efficient means for the plague bacillus to adapt to new ecological niches. Yersinia pestis was, furthermore, recently shown to be able to acquire antibiotic resistance plasmids under natural conditions [56,57], probably during its transit in the flea midgut [58]. Of great concern is the recent observation of the presence of multidrug-resistant plasmids, almost identical to those acquired by Y. pestis, in a variety of enterobacteria isolated from retail meat products in the United States [59]. This bacterial reservoir of mobile resistance determinants is probably widespread globally and has the potential to disseminate to human and zoonotic bacterial pathogens, including Y. pestis. Obviously, the emergence and spread of multi-resistant strains of Y. pestis would represent a major threat to human health.

Finally, we should not overlook the fact that plague has been weaponised throughout history, from catapulting corpses over city walls, to dropping infected fleas from airplanes, to refined modern aerosol formulations [60,61]. The weaponisation research on plague carried out from before World War II until the 1990s fuelled a fear of biological warfare that may actually have stimulated research into surveillance and response strategies. More recently, however, fear of small-scale bioterrorism and the desire of governmental authorities to more fully control all access to plague materials risks stifling the research on plague ecology, epidemiology, and pathophysiology that is required to improve its clinical management in endemic areas. Terrorist use of an aerosol released in a confined space could result in significant mortality and widespread panic [60,61], and no one would wish plague weaponisation knowledge and material to fall into terrorist hands. However, the need for scientifically sound studies on the dynamics of infection, transmission, outbreak management, and improved surveillance and monitoring systems has never been greater.

Plague may not match the so-called “big three” diseases (malaria, HIV/AIDS, tuberculosis; see for example [62]) in numbers of current cases, but it far exceeds them in pathogenicity and rapid spread under the right conditions. It is easy to forget plague in the 21st century, seeing it as a historical curiosity. But in our opinion, plague should not be relegated to the sidelines. It remains a poorly understood threat that we cannot afford to ignore.

## Supporting Information

Alternative Language Text S1 Translation of article into French by EB (303 KB PDF)

Alternative Language Text S2 Translation of article into Russian by BBA (356 KB PDF)

## Abbreviations

DRC: Democratic Republic of Congo
WHO: World Health Organization
