# Supplementary material for: Plague: Past, Present, and Future
Source: PLoS Med. 2008 Jan 15;5(1):e3. doi: 10.1371/journal.pmed.0050003 (PMC2194748; doi:10.1371/journal.pmed.0050003)
Supplement: Supplementary file 1 [file pmed.0050003.sd001.pdf]

(16.09.06; French translation by Eric Bertherat)
PLoS Medicine “Neglected Diseases”

La Peste: Passé, Présent et Futur

Nils Chr. Stenseth[1], Bakyt B Atshabar[2], Mike Begon[3], Steven R. Belmain[4], Eric Bertherat[5], Elisabeth Carniel[6], Kenneth L. Gage[7], Herwig Leirs[8,9] & Lila Rahalison[10]

[1] Centre for Ecological and Evolutionary Synthesis (CEES), Department of Biology, University of Oslo, PO Box 1066 Blindern, 0316 Oslo, Norway
[2] Kazakh Scientific Centre for Quarantine and Zoonotic Diseases, Almaty, Kazakhstan
[3] School of Biological Sciences, Biosciences Building, University of Liverpool, Liverpool L69 7ZB, UK
[4] Natural Resources Institute, University of Greenwich, Central Avenue, Chatham Maritime, Kent ME4 4TB, UK
[5] Epidemic Readiness and Interventions, Department of Epidemic and Pandemic Alert and Response, World Health Organization, 1211 Geneva 27, Switzerland
[6] Yersinia Research Unit, Institut Pasteur, 28 rue du Dr.Roux, 75724 Paris CEDEX 15, France
[7] Flea-born Diseases Laboratory, Centers for Disease Control and Prevention (CDC), Fort Collins, USA
[8] Evolutionary Biology Group, University of Antwerp, Groenenborgerlaan 171, BE-2020 Antwerpen, Belgium
[9] Danish Pest Infestation Laboratory, Danish Institute of Agricultural Sciences, Department of Integrated Pest Management, Skovbrynet 14, DK-2800 Kongens Lyngby, Denmark
[10] Laboratoire Central de la Peste, Institut Pasteur de Madagascar, BP1274, Antananarivo, Madagascar.

[†] Corresponding author: N.C. Stenseth: phone: +47-22854584/4400; fax: +47-22854001; e-mail: n.c.stenseth@bio.uio.no

L'expérience récente du SRAS [1] et de la grippe aviaire a montré que la réaction du public et des politiques aux menaces de nouvelles anthropozoonoses peut friser l'hystérie. Ceci peut nous faire trop facilement oublier des maladies d'origine animale plus classiques telles que la peste (Encadré 1).

Les défis persistants que soulève la peste ont été abordés lors de trois récentes réunions internationales. Une réunion multi-disciplinaire sur la peste du présent, du passé et du futur s'est tenue à l'Académie des Sciences et Lettres d'Oslo, Norvège (http://www.cees.no/oslo-plague-meeting). Un atelier sur le thème de la Peste Noire du Moyen Age et la peste actuelle a été organisé par le *Centre Wellcome Trust pour l'Histoire de la Médecine* (University College, Londres; http://www.ucl.ac.uk/histmed/). L'OMS a été à l'origine d'une réunion d'experts à Antananarivo, Madagascar pour mettre à jour les recommandations en matière de définition de cas, de méthodes diagnostiques, de vaccins et traitement ainsi que de stratégie de contrôle des réservoirs et des vecteurs (http://www.who.int/csr/disease/plague/interregionalmeeting2006/en/index.html).

Les principales conclusions communes à ces trois réunions ont été les suivantes: (i) il faut souligner que le bacille pesteux (*Yersinia pestis*) continue de causer plusieurs milliers de cas humains chaque année [2,3] (Fig. 1); (ii) le risque tel qu'il est perçu localement dépasse largement le risque objectif calculé sur le seul nombre de cas [2]; (iii) le changement de climat pourrait augmenter le risque de survenue d'épidémies de peste dans les zones endémiques et de nouvelles zones endémiques pourraient apparaître [2,4]; (iv) on sait étonnamment peu de choses de la dynamique de la peste dans ses réservoirs naturels et par conséquent de l'évolution du risque pour l'Homme [5]; et, de fait, (v) la peste doit être prise au sérieux par la communauté internationale bien plus qu'elle ne semble l'être actuellement.

Le Cycle Éco-Épidémiologique de la Peste

Le bacille pesteux est à l'origine d'une infection sérieuse et d'évolution rapide, à forte létalité dans sa forme bubonique (40-70%) en l'absence d'un traitement antibiotique précoce ; la peste pulmonaire comme le peste septicémique sont quasiment toujours fatales en l'absence de traitement. Pour ces

raisons, *Y. pestis* est considérée comme une des bactéries les plus pathogènes pour l'Homme. Elle est transmise par des puces, alors que les deux autres espèces de *Yersinia* connues pour être pathogènes pour l'Homme (*Y. enterocolitica* and *Y. pseudotuberculosis*) sont transmises par voie oro-fécale et provoquent des symptômes digestifs d'intensité modérée. On pense que *Y. pestis* est un clone de *Y. pseudotuberculosis* qui a émergé il y a 1500 à 20 000 ans [6,7]. Cette différenciation a été caractérisée par l'acquisition de quelques éléments génétiques, et plus particulièrement de deux plasmides qui jouent un rôle dans la transmission par la puce [8,9]. L'exceptionnelle pathogénicité de *Y. pestis* par comparaison avec les espèces entéropathogènes peut être expliquée par son nouveau mode de transmission. En effet, les seuls moyens pour cette bactérie d'être transférée vers un nouvel hôte est de causer une septicémie, permettant ainsi à la bactérie d'être présente dans le flux sanguin et d'être ingurgitée par la puce durant son repas sanguin [10].

Peu après la découverte du bacille pesteux par Yersin [11], il est devenu clair que les épidémies urbaines étaient liées à la transmission parmi les rats commensaux et leurs puces. Dans ce scénario classique de peste urbaine, des rats infectés (transportés par exemple par des bateaux), arrivent dans une nouvelle ville et transmettent l'infection aux rats domestiques locaux et à leurs puces. Occasionnellement, des êtres humains développent la forme pulmonaire de la peste qui est ensuite directement transmise d'Homme à Homme par les gouttelettes émises lors de la toux.

L'épidémiologie de la peste, cependant, est beaucoup plus compliquée que ce que peut suggérer ce scénario de peste urbaine, impliquant plus probablement plusieurs autres voies de transmission (Encadré 2). Ceci nécessite de reconsidérer l'écologie de la peste.

Le Passé

La peste a été à l'origine d'au moins trois pandémies majeures. La première ("peste de Justinien") a affecté le pourtour méditerranéen au VIème siècle, la seconde ("Peste Noire") a émergé en Europe au XIVème siècle et s'est manifestée de façon récurrente durant plus de 300 ans, la troisième enfin est apparue en Asie Centrale au milieu du XIXème siècle et a essaimé dans le monde entier. Chacune de ces pandémies aurait été causée par un biovar différent de *Y. pestis*, respectivement Antiqua (encore présent en Afrique et en Asie Centrale), Medievalis (aujourd'hui limité à l'Asie Centrale) et Orientalis (quasiment présent dans le monde entier) [12,13].

La Peste Noire a décimé l'Europe médiévale et eu un impact majeur sur le développement socio-économique du continent, la culture, l'art, la religion et la politique [14,15]. Plusieurs spécialistes de haut niveau ont remis en question la responsabilité effective de *Y. pestis* dans la Peste Noire [16,17]. Les partisans de chaque thèse se sont rencontrés, tant à la réunion d'Oslo qu'à celle de Londres. Il a été généralement reconnu que l'épidémiologie de la Peste Noire, telle que reflétée par les documents historiques, n'a pas toujours correspondu au cycle "classique" rat-puce-Homme. Cependant, les symptômes qui ont été rapportés étaient très similaires à chaque pandémie, et l'expérience internationale de la peste moderne, représentée à la réunion d'Oslo, a mis en évidence que l'épidémiologie "classique" de la peste n'en est qu'une parmi d'autres, et peut être même pas la plus typique [18]. La découverte de matériel génétique de *Y. pestis* chez des victimes de la Peste Noire inhumées dans des tombes médiévales [19] renforce l'hypothèse que *Y. pestis* était bien l'agent causal de la seconde pandémie.

Le Présent

Son importance historique a conduit la peste à être souvent considérée comme un problème du passé. Cependant, elle reste une menace actuelle dans beaucoup de régions du monde (Fig. 1a), particulièrement en Afrique ou tant le nombre de cas de peste (Fig 1b) que le nombre de pays déclarant des cas (Fig 1c) a augmenté durant les dernières décennies. A la suite de la réapparition de la peste dans les années 90 dans plusieurs pays, la peste a été qualifiée de maladie ré-émergente [20,21].

La peste est endémique dans de nombreuses espèces de rongeurs sauvages dans le monde, vivant dans des habitats naturels très différents, avec des rats commensaux jouant seulement un rôle d'hôtes intermédiaires, transportant la peste du réservoir selvatique à la population humaine. De nombreuses autres voies de transmission de l'animal à l'Homme ont été documentées. La peste humaine peut être contractée par (i) piqûre de puces d'espèces de rongeurs sauvages en contexte rural (ex. dans le sud-ouest des Etats-Unis [22,23]) ou de rongeurs commensaux qui se déplacent librement entre les villages et les habitats forestiers occupés par les hôtes réservoirs (ex. Tanzanie): déplacements devenant plus fréquents quand l'activité humaine a fragmenté la forêt [24]; (ii) consommation d'animaux infectés comme des cobayes au Pérou et en Equateur [25,26] ou de chameaux qui contractent la maladie à partir de puces de rongeurs en Asie Centrale et au Moyen-Orient [27-31]; ou (iii) manipulation de chats infectés après consommation de rongeurs infectés eux-mêmes en Afrique ou aux Etats-Unis [32-34]. La transmission interhumaine survient aussi, soit directement par les gouttelettes de Pflügge ou indirectement par les piqûres de puces [35-37].

Durant les 20 dernières années, 1 000 à 5 000 cas humains et 100 à 200 décès ont été rapportés à l'OMS chaque année [38]. Cependant, du fait des faibles capacités diagnostiques et de la sous-déclaration, le nombre de cas est certainement beaucoup plus élevé. Au fil des ans, il y a eu un déplacement majeur des cas de l'Asie vers l'Afrique (Fig 1b), avec plus de 90% de tous les cas et décès dans les cinq dernières années survenant à Madagascar, en Tanzanie, au Mozambique, Malawi, Ouganda et République Démocratique du Congo (RDC). La plupart sont des cas de peste bubonique contractés par contact avec des rongeurs et des puces infectés. Cependant, des épidémies de peste pulmonaire surviennent encore, la plus récente en RDC entre mai et juillet 2006, avec plus de 200 cas suspects (http://www.who.int/csr/don/2006_06_14/). En décembre 2004 il y avait déjà eu une épidémie de peste pulmonaire dans un camp de mineurs en RDC, probablement importée par une personne infectée en provenance d'une zone endémique. Un cas pulmonaire est même parvenu à Kisangani, une ville importante à plusieurs centaines de kilomètres [39,40]. Même une réponse médicale précoce peut être insuffisante pour empêcher une extension rapide de la maladie à de grandes distances avant qu'elle ne soit détectée et prise en charge.

L' Afrique est particulièrement exposée pour de nombreuses raisons. Les communautés rurales pauvres vivent habituellement en contact étroit avec les rongeurs. Les rongeurs sont largement chassés et consommés dans beaucoup de zones endémiques de peste. Les superstitions, le manque de moyens et l'éloignement des centres de santé conduisent souvent à retarder le recours à la prise en charge et au traitement. Le système de santé publique dans beaucoup de régions d'Afrique est mal organisé et équipé, et crises politiques comme désorganisation sociale empêchent toute amélioration. Finalement, les changements anthropogéniques du paysage et des modes de déplacement humain favorisent considérablement les contacts entre le réservoir sauvage de peste et les rongeurs péri-domestiques, et entre les populations des zones endémiques et celles des zones non encore affectées.

Un Regard vers le Futur

La peste ne peut pas être éradiquée du fait de sa large diffusion parmi les rongeurs sauvages. Ainsi il est indispensable de comprendre comment les risques chez l'Homme sont affectés par la dynamique de ces réservoirs sauvages. Par exemple, il a été montré que la probabilité d'une épizootie de peste parmi les rongeurs d'Amérique du Nord et d'Asie Centrale, et le risque résultant pour la population humaine, était liée au climat [41,42]. Une analyse récente des données du Kazakhstan [43] montre que des printemps plus chauds et des étés plus humides augmentent la prévalence de la peste chez son hôte principal, la grande gerbille. De telles conditions environnementales semblent avoir prévalu également quand sont survenues les seconde et troisième pandémies [44] – conditions qui pourraient devenir plus habituelles dans le futur [45].

Bien que le nombre de cas de peste humaine soit relativement faible, ce serait une erreur de négliger sa menace sur l'humanité, du fait de la communicabilité inhérente à la maladie, de sa diffusion

et de son évolution clinique rapides ainsi que de sa forte létalité en l'absence de traitement. Une épidémie de peste peut aussi causer une vaste panique, comme en Inde en 1994 lorsqu'une épidémie de relativement faible ampleur, avec 50 décès, s'est déclarée dans la ville de Surat [46]. Cette panique entraina un effondrement du tourisme et du commerce d'importance nationale, avec un coût estimé à 600 millions de dollars [47].

Les épidémies sont habituellement contrôlées comme on traite des incendies. Des équipes pénètrent dans la zone infectée pour éliminer les puces avec des insecticides, pour traiter les cas humains et distribuer une chimioprophylaxie aux personnes exposées. Beaucoup d'experts ont soutenu que cette approche de gestion de crise n'est pas suffisante, l'épidémie étant probablement sur le déclin quand les mesures sont prises. Des décisions documentées et préparées avant que les épidémies ne surviennent seraient certainement plus viables et financièrement bénéfiques. Il y a eu quelques progrès récents, tels que le développement de tests de diagnostic rapide [48], quelques remises en cause de dogmes à propos de la dynamique de la peste selvatique aux Etats-Unis [49] et en Asie Centrale [50], ainsi que l'identification de seuils prédictifs critiques de l'abondance des rongeurs au Kazakhstan [51]. Ce qu'il faut retenir, pourtant, est notre méconnaissance de cette maladie particulière même dans les foyers les mieux étudiés, particulièrement en Afrique: souvent, nous ne connaissons même pas le réservoir naturel.

La capacité du bacille pesteux de constituer des foyers permanents dans des conditions écologiques très variées, atteste de son extraordinaire adaptabilité. Durant son émergence en Asie Centrale, *Y. pestis* a accumulé des copies de séquences d'insertion ayant rendu son génome hautement plastique [52]. La garnde capacité de subir des réarrangements génomiques peut donc être un moyen efficace pour le bacille pesteux de s'adapter à de nouvelles niches écologiques. *Y. pestis*, de plus, s'est montré capable d'acquérir des plasmides de résistance aux antibiotiques en conditions naturelles [53,54], probablement durant son transit dans l'œsophage de la puce [55]. De façon évidente, l'émergence et l'expansion de souches multi-résistantes de *Y. pestis* constitueraient une menace majeure pour la santé humaine.

Enfin, nous ne devons pas oublier que la peste a été utilisée comme arme tout au long de l'Histoire, depuis les corps catapultés par-dessus les murs des places-fortes et les puces infectées larguées des avions, jusqu'au formulations modernes d'aérosols [56,57]. Des recherches portant sur l'utilisation délibérée de la peste, conduites depuis les années 30 jusqu'aux années 90, ont entretenu une crainte de conflit biologique qui peut en fait avoir stimulé la recherche vers des stratégies de surveillance et de réponse. Plus récemment cependant, les craintes d'un bioterrorisme à petite échelle, et le souhait des autorités gouvernementales de contrôler plus complètement tout accès à du matériel pesteux, a augmenté le risque d'étouffer la recherche sur l'écologie, l'épidémiologie et la physiopathologie de la peste. Celle-ci est pourtant indispensable pour améliorer la prise en charge de la maladie dans les régions endémiques. L'utilisation à des fins terroristes d'un aérosol dans un espace confiné pourrait entrainer une mortalité significative et une vaste panique [56,57], et personne ne souhaiterait que la connaissance en matière d'usage délibéré et le matériel correspondant ne tombe entre les mains de terroristes. Cependant, le besoin d'études scientifiques approfondies sur la dynamique de l'infection et de la transmission, la prise en charge des épidémies et l'amélioration des systèmes de surveillance et de suivi n'a jamais été aussi grand.

La peste ne peut sans doute pas rivaliser avec les grands fléaux infectieux que sont le paludisme, le SIDA et la tuberculose [58] en termes de nombre de cas, mais elle les dépasse de loin en termes de pathogénicité et de rapidité de diffusion dans des conditions favorables. Il est tentant d'oublier la peste au XXIème siècle, et de la considérer comme une curiosité historique. Mais selon nous la peste ne doit pas être reléguée aux oubliettes. Elle reste une menace mal connue que l'on ne peut pas se permettre d'ignorer.

References

1. McLean, A.R., May, R.M., Pattison, J. & Weiss, R.A. (2005) *SARS. A case study in emerging infections*. Oxford University Press, Oxford.
2. World Health Organisation (2003) Plague. *Weekly Epidemiological Record*, **78**, 253-260.
3. World Health Organisation (2005) Plague. *Weekly Epidemiological Record*, **80**, 138-140.
4. Stapp, P., Antolin, M.F. & Ball, M. (2004) Patterns of extinction in prairie dog metapopulations: plague outbreaks follow El Nino events. *Frontiers in Ecology and the Environment* **2**, 235–240.
5. Gage, K.L. & Kosoy, M.Y. (2005) Natural history of plague: perspectives from more than a century of research. *Annual Review of Entomology* **50**, 505–528.
6. Achtman, M., Zurth, K., Morelli, G., Torrea, G., Guiyoule, A. & Carniel, E. (1999) *Yersinia pestis*, the cause of plague, is a recently emerged clone of *Yersinia pseudotuberculosis*. *Proceedings of National Academy of Science, Washington* **96**, 14043-14048.
7. Achtman, M., Morelli, G., Zhu, P., Wirth, T., Diehl, I., Kusecek, B., Vogler, A.J., Wagner, D.M., Allender, C.J., Easterday, W.R., Chenal-Francisque, V., Worsham, P., Thomson, N.R., Parkhill, J., Lindler, L.E., Carniel, E., & Keim, P. (2004) Microevolution and history of the plague bacillus, *Yersinia pestis*. *Proceedings of National Academy of Science, Washington* **101**, 17837–17842.
8. Sodeinde, O. A. et al. (1992) A surface protease and the invasive character of plague. *Science* **258**, 1004-1007.
9. Hinnebusch, B. J. *et al.* (2002) Role of *Yersinia* murine toxin in survival of *Y. pestis* in the midgut of the flea vector. *Science* **296**, 733-735.
10. Carniel, E. (2003) Evolution of pathogenic Yersinia, some lights in the dark. *Advances in Experimental Medical Biology* **529**, 3-12.
11. Yersin, A. (1894) La peste bubonique à Hong-Kong. *Ann. Inst. Pasteur* **2**, 428-430.
12. Devignat, R. (1951) Variétés de l'espèce *Pasteurella pestis*. Nouvelle hypothèse. *Bulletin de l'Organisation Mondiale de la Santé* **4**, 247-263.
13. Guiyoule, A. Grimont, F., Iteman, I., Grimont, P.A.D., Lefevre, M. & Carniel, E. (1994) Plague pandemics investigated by ribotyping of *Yersinia pestis* strains. *Journal of Clinical Microbiology* **32**, 634-641.
14. Twigg, G. (1984) *The Black Death: a biological reappraisal*. Batsform Academic and Educational, London.
15. Ziegler, P. (1969) *The Black Death*. Alan Sutton Publishing Inc., Wolfeboro Falls, NH.
16. Scott, S. & Duncan, C.J. (2001) *Biology of Plagues: Evidence from Historical Populations.* Cambridge University Press, Cambridge.
17. Cohn, Jr., Samuel K. (2002) *The Black Death Transformed.* Arnold, London.
18. Drancourt, M. (2006) *Yersinia pestis* as a telluric, human ectoparasite-borne organism. *Lancet Infectious Disease* **6**, 234-241.
19. Raoult, D., Aboudharam, G., Crubézy, E., Larrouy, G., Ludes, B. & Drancourt, M. (2000) Molecular identification by "suicide PCR" of *Yersinia pestis* as the agent of Medieval Black Death. *Proceedings of National Academy of Science, Washington* **97**, 12800–12803.
20. Schrag, S. J. & Wiener, P. (1995) Emerging infectious diseases: what are the relative roles of ecology and evolution? *Trends in Ecology and Evolution* **10**, 319-324.
21. Duplantier, J. M., Duchemin, J. B., Chanteau, S. & Carniel, E. (2005) From the recent lessons of the Malagasy foci towards a global understanding of the factors involved in plague reemergence. *Veterinary Research* **36**, 437-453.
22. Gage, K.L., Ostfeld, R.S. & Olson, J.G. (1995) Nonviral vector-borne zoonoses associate with mammals in the United States. *Journal of Mammalogy* **76**, 695-715.

23. Levy, C.E, & Gage, K.L (1999). Plague in the United States 1995-1996. *Infections in Medicine* **16**, 54-64.
24. Kaoneka, A.R.S & Solberg, B. (1994) Forestry-related land use in the West Usambara mountains, Tanzania. *Agriculture, Ecosystems and Environment*. **49**, 207-215.
25. Gabastou J-M, Proano J, Vimos A, Jaramillo G, Hayes E, Gage K, Chu M, Guarner J, Zaki S, Bowers J, Guillemard C, Tamayo H, and Ruiz A. An outbreak of plague including cases with pneumonic infection, Ecuador, 1998. *Transactions of the Royal Society of Tropical Medicine and Hygiene* **94**, 387-391 (2000).
26. Ruiz A. (2001) Plague in the Americas. *Emerging Infectious Diseases* **7**, (Supplement) 539-540.
27. Fedorov V.N. (1960) Plagues in camels and its prevention in the USSR *Bulletin of WHO* **23**, 275-281.
28. Christie, A.B., Chen, T.H.& Elberg, S.S.(1980) Plague in camels and goats: their role in human epidemics. *Journal of Infectious Disease* **141**, 724-726.
29. Atshabar B. B., Aikimbaev A.M., Aubakirov S.A., Suleimenov B.M. et al. (2000) Epizootologic and social basis for plague human infection in 1999 in Kazakhstan and their clinical-epidemiologic characteristics. *Problems of the most dangerous infections,* Saratov, 14-21.
30. Arbaji, A, Kharabsheh, S., Al Azab, S., Al Kayed, M., Amr, Z.S., Baker, M.A. & Chu, M.C. (2005) A 12-case outbreak of pharyngeal plague following the consumption of camel meat, in north-eastern Jordan. *Annals of Tropical Medicine and Parasitology* **99**, 789-793.
31. Bin Saeed, A.A., Al-Hamdan, N.A. (2005) Fontaine RE. Plague from eating raw camel liver. *Emerging Infect Disease* **11**, 1456-1457.
32. Isaacson, M. (1973) Unusual cases of plague in southern Africa. *South African Medical Journal* **47**, 2109-2113.
33. Doll, J. M., Seitz, P. S., Ettestad, P., Bucholtz, A. L., Davis, T., & Gage, K.L. (1994) Cat Transmitted Fatal Pneumonic Plague in a Person Who Travelled from Colorado to Arizona. *American Journal of Tropical Medicine and Hygiene* **51**, 109-114.
34. Gage, K.L., Dennis, D.T., Orloski, K.A., Ettestad, P.J., Brown, T.L., Reynolds, P.J., Pape, W.J., Fritz, C.L., Carter, L.G. & Stein, J.D. (2000) Cases of cat-associated human plague in the Western US, 1977-1998. *Clinical Infectious Diseases* **30**, 893-900.
35. Pollitzer R. (1954) Plague. *World Health Organization Monograph Series* **22**, 1-698.
36. Pollitzer, R. (1960) A review of recent literature on plague. *Bulletin of WHO* **23**, 313-400.
37. Blanc G. (1956) Une opinion non conformiste sur le mode de transmission de la peste. *Revue d'Hygiéne et de Médecine Sociale* **4**, 532-562.
38. World Health Organisation (2004) Human Plague in 2002 and 2003. *Weekly Epidemiological Record* **79(33)**, 301-306.
39. Bertherat, E., Lamine, K.M., Formenty, P., Thullier, P., Mondonge, V., Mitifu, A. & Rahalison, L. (2005) Epidémie de peste pulmonaire dans un camp minier de la République Démocratique du Congo: le réveil brutal d’un vieux fléau. *Médecine Tropicale* **65**, 511-514.
40. Bertherat, E., Kone, M., Formenty, P., Thullier, P., Mondonge, V., Rabarijaona, L., Shako, J.C., Ekwanzala, F., Mitifu, A., Kayembe, J., Mossoko, M., Arntzen, L. & Rahalison, L. (2006) Emergence of plague in democratic Republic of Congo: a large pneumonic outbreak erupts in a diamond mine. *International Conference on Emerging Infectious Diseases. Atlanta , Georgia, USA, March 19-22, 2006.*
41. Parmenter, R.R., Yadav, E.P., Parmenter, C.A., Ettestad, P.J. & Gage, K.L. (1999) Incidence of plague associated with increased winter-spring precipitation in Mexico. *American Journal of Tropical Medicine and Hygiene* **61**, 814-821.
42. Enscore, R.E., Biggerstaff, B.J., Brown, T.L., Fulgham, R.F., Reynolds, P.J., Engelthaler, D.M., Levy, C.E., Parmenter, R.R., Montenieri, J.A., Cheek, J.E., Grinnell, R.K., Ettestad, P.J. &

Gage, K.L. (2002) Modeling relationships between climate and the frequency of human plague cases in the southwestern United States, 1960–1997. *American Journal of Tropical Medicine and Hygiene* **66**, 186–196.
43. Stenseth, N.C., Samia, N.I., Viljugrein, H., Kausrud, K., Begon, M. Davis, S., Leirs, H., Dubyanskiy, V.M, Esper, J., Ageyev, V.S., Klassovskiy, N.L., Pole, S.B. & Chan, K.-C. (2006) Plague Dynamics are driven by Climate Variation. *Proceedings of National Academy of Science, Washington* **103**, 13110-13115..
44. Treydte, K., Schleser, G.H., Helle, G., Frank, D.L., Winiger, M., Haug, G.H. & Esper, J. (2006) Twentieth century as the wettest period in northern Pakistan over the past century. *Nature* **440**, 1179-1182.
45. IPCC. *Climate Change 2001: The Scientific Basis. Contribution of Working Group I to the Third Assessment Report of the Intergovernmental Panel on Climate Change* [Houghton, J.T., Ding, Y, Griggs, D.J., Noguer, M., van der Linden, P.J., Dai, X., Maskell, K. & Johnson, C.A. (eds.)]. Cambridge University Press, Cambridge, United Kingdom and New York, NY, USA, 881pp.
46. Ganapati, M. (1995) India's pneumonic plague outbreak continues to baffle. *British Medical Journal* **311**, 706.
47. Fritz, C.L., Dennis, D.T., Tipple, M.A., Campbell, G.L., McCance, C.R., & Gubler, D.J. (1996) Surveillance for Pneumonic Plague in the United States During an International Emergency: A Model for Control of Imported Emerging Diseases. *Emerging Infectious Diseases* **2**, 30-36.
48. Chanteau S., Rahalison L., Ralafiarisoa L., Foulon F., Ratsitorahina M., Ratsifasoamanana L., Carnie E. & Nato F. (2003) Development and testing of a rapid diagnostic test for bubonic and pneumonic plague. *The Lancet,* **361**, 211-216.
49. Webb, C.T., Brooks, C.P., Gage, K.L. & Antolin, M.V. (2006) Classic flea-born transmission does not drive plague epizootics in prairie dogs. *Proceedings of National Academy of Science, Washington* **103**, 6236-6241.
50. Begon, M., Klassovskiy, N., Ageyev, V., Suleimenov, B., Atshabar, B. & Bennett, M. (2006) Epizootiologic parameters for plague in Kazakhstan. *Emerging Infectious Diseases* **12**, 268-273.
51. Davis, S., Begon, M., De Bruyn, L., Ageyev, V., Viljugrein, H., Stenseth, N. & Leirs, H. (2004) Predictive Thresholds for Plague in Kazakhstan. *Science* **304**, 736–738.
52. Parkhill, J., Wren, B.W., Thomson, N.R. *et al.* (2001) Genome sequence of *Yersinia pestis*, the causative agent of plague. *Nature* **413**, 523-527.
53. Galimand, M., Guiyoule, A., Gerbaud, G., Rasoamanana, B., Chanteau, S., Carniel, E. & Courvalin, P. (1997) Multidrug resistance in *Yersinia pestis* mediated by a transferable plasmid. *New England. Journal of Medicine* **337**, 677-680.
54. Guiyoule, A. Gerbaud, G., Buchrieser, C., Galimand, M., Rahalison, L., Chanteau, S., Courvalin, P. & Carniel, E. (2001) Transferable plasmid-mediated resistance to streptomycin in a clinical isolate of *Yersinia pestis*. *Emerging Infectious Disease* **7**, 43-48.
55. Hinnebusch, B. J., Rosso, M. L., Schwan, T. G. & Carniel, E. (2002) High-frequency conjugative transfer of antibiotic resistance genes to *Yersinia pestis* in the flea midgut. *Molecular Microbiology* **46**, 349-354.
56. Inglesby, T.V., Dennis, D.T., Henderson, D.A., Bartlett, J.G., Ascher, M.S., Eitzen, E., Fine, A.D., Friedlander, A.M., Hauer, J., Koerner, J.F., Layton, M., McDade, J., Osterholm, M.T., O'Toole, T. Parker, G., Perl, T.M. Russell, P.K., Schoch-Spana, M., Tonat, K. (2000) Plague as a Biological Weapon: Medical and Public Health Management. *Journal of the American Medical Association* **283**, 2281-2290.
57. Koirala, J. (2006) Plague: disease, management, and recognition of act of terrorism. *Infectious disease clinics of North America* **20**, 273-287.

58. Hotez, P.J., Molyneux, D.H., Fenwick, A., Ottesen, E., Sachs, S.E. & Sachs, J.D. (2006) Incorporating a reapid-impact package for neglected tropical diseases with programs for HIV/AIDS, Tuberculosis, and Malaria. *PloS Medicine* **3**, 576-584.
59. Simond, P.L. (1898) La propagation de la peste. *Annales de l'Institut Pasteur* **12**, 625-687.
60. Baltazard, M., Karimi, Y., Eftekhari, M., Chamsa, M. & Mollaret, H. H. (1963) La conservation interépizootique de la peste en foyer invétéré hypothèses de travail. *Bulletin de la Société de Pathologie Exotique* **56**, 1230-1241.

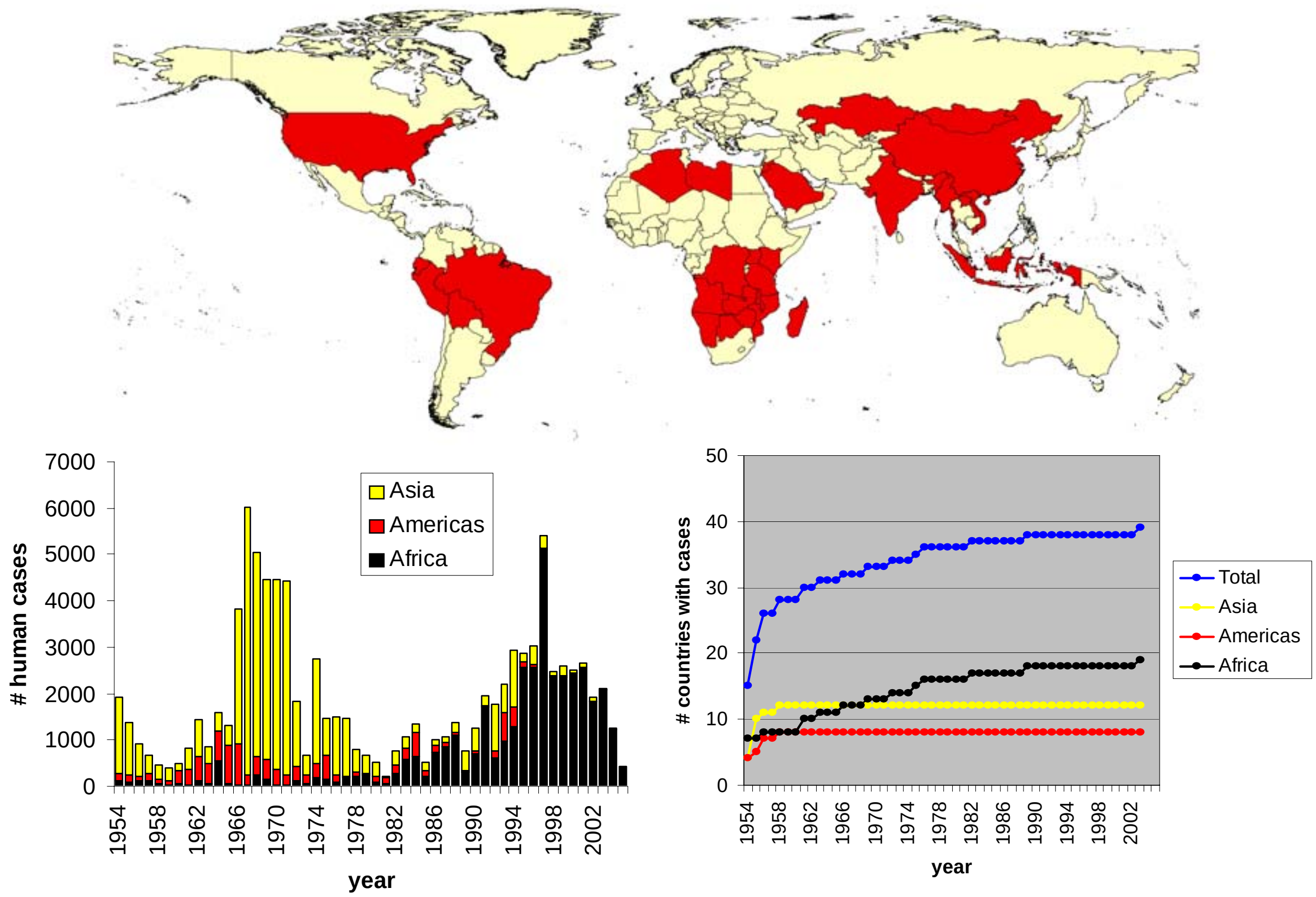

Figure 1: Distribution mondiale de la peste. (a) Carte des pays rapportant une présence de la peste dans les réservoirs sauvages (rouge) (d'après [3]) (Pour les Etats-Unis on ne montre que ce qui est situé sous le 50°N); (b) Nombre annuel des cas de peste sur les différents continents, rapportés à l'OMS pendant la période 1954-2005; (c) Nombre cumulatif des pays qui ont rapporté une présence de la peste depuis 1954.

Box 1: The Plague

The causative bacterium (*Yersinia pestis*) of plague was discovered by Yersin in 1894 [11] (see also [59]). Case-fatality ratio varies from 30 to 100%, if left untreated. Plague is endemic in many countries, in the Americas, Asia and Africa, currently with more than 90% of the cases being reported from the latter continent.

Clinical presentation: After an incubation period of 3-7 days, patients typically experience a sudden onset of fever, chills, headaches, body aches, weakness, vomiting and nausea. Clinical plague infection manifests itself in 3 forms, depending on the route of infection: bubonic, septicaemic and pneumonic. The bubonic form is the most common form resulting from the bite of an infected flea. The pneumonic form initially is directly transmitted from human to human via inhalation of infected respiratory droplets.

Treatment: Rapid diagnosis and treatment are essential to reduce complications and fatality. Streptomycin, tetracyclines and sulfonamides remain the main antibiotics recommended for plague treatment. Gentamicin and fluoroquinolones mayrepresent alternatives when the above antibiotics are not available. Pneumonic plague patients must be isolated to avoid respiratory transmission.

Some steps forward: Biological diagnosis of plague remains a challenge because most human cases appear in remote areas with scarce laboratory resources. So far, the main confirmation techniques were based on the isoltation of *Y. pestis* (requiring a minimum of 4 days). The recent development of rapid diagnostic tests, considered now as a confirmation method in endemic areas, opens new perspectives in term of surveillance and case management.

Box 2: The plague bacterium within the ecological web

Maintenance of plague foci depends on a whole suite of rodent hosts and their associated fleas (Fig 2 (cycle a)). Under favourable conditions, the plague bacillus might survive in the environment, essentially in rodent burrows [60].When an infected flea happens to feed on a commensal rodent, the cycle continues in the latter (Fig 2 (cycle b)). As commensal rodents die, their fleas are forced to move to alternative hosts, e.g., humans. If humans develop pneumonic plague, the infection may transmit from person-to-person through respiratory droplets spread by coughing (Fig 2 (cycle c)). Humans may also become infected through handling of infected animals (or meat), including rodents, camels, or cats. Cats also can develop pneumonic plague, passing their infection to their owners through coughing. There is, finally, evidence that the human flea, *Pulex irritans*, can be involved in human-to-human transmission [37]. Mammal predators, birds of prey and other birds that use rodent burrows for nesting may move over larger areas than the rodents themselves, spreading the infection over longer distances. Also, infected commensal rats or humans may travel over long distances.

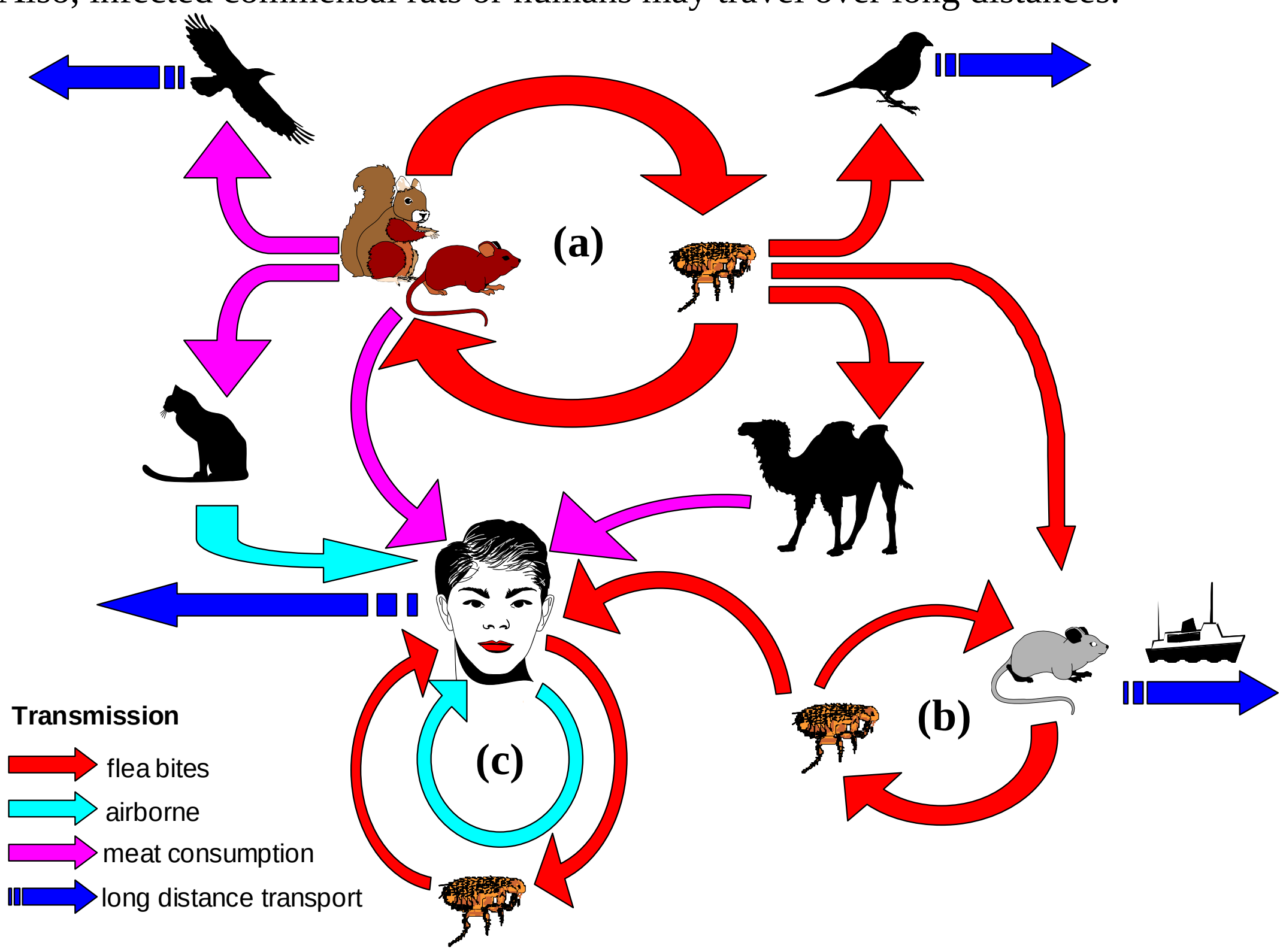

Figure 2: Possible transmission pathways for the plague agent, *Y. pestis*, including sylvatic rodent-flea cycles (a), the commensal rodent-flea cycles (b) and the pneumonic transmission in humans (c). The colour of the arrows indicates the mechanism (flea bites, air particles, meat consumption) through which the bacteria are transferred from one host to another. Dark blue arrows indicate ways in which plague can move to other areas.
