# Supplementary material for: Plague: Past, Present, and Future
Source: PLoS Med. 2008 Jan 15;5(1):e3. doi: 10.1371/journal.pmed.0050003 (PMC2194748; doi:10.1371/journal.pmed.0050003)
Supplement: Supplementary file 2 [file pmed.0050003.sd002.pdf]

(15.09.06; French translation by Bakyt B Atshabar)
PLoS Medicine “Neglected Diseases”

Чума: Прошлое, Настоящее и Будущее

Nils Chr. Stenseth[1], Bakyt B Atshabar[2], Mike Begon[3], Steven R. Belmain[4], Eric Bertherat[5], Elisabeth Carniel[6], Kenneth L. Gage[7], Herwig Leirs[8,9] & Lila Rahalison[10]

[1] Centre for Ecological and Evolutionary Synthesis (CEES), Department of Biology, University of Oslo, PO Box 1066 Blindern, 0316 Oslo, Norway
[2] Kazakh Scientific Centre for Quarantine and Zoonotic Diseases, Almaty, Kazakhstan
[3] School of Biological Sciences, Biosciences Building, University of Liverpool, Liverpool L69 7ZB, UK
[4] Natural Resources Institute, University of Greenwich, Central Avenue, Chatham Maritime, Kent ME4 4TB, UK
[5] Epidemic Readiness and Interventions, Department of Epidemic and Pandemic Alert and Response, World Health Organization, 1211 Geneva 27, Switzerland
[6] Yersinia Research Unit, Institut Pasteur, 28 rue du Dr.Roux, 75724 Paris CEDEX 15, France
[7] Flea-born Diseases Laboratory, Centers for Disease Control and Prevention (CDC), Fort Collins, USA
[8] Evolutionary Biology Group, University of Antwerp, Groenenborgerlaan 171, BE-2020 Antwerpen, Belgium
[9] Danish Pest Infestation Laboratory, Danish Institute of Agricultural Sciences, Department of Integrated Pest Management, Skovbrynet 14, DK-2800 Kongens Lyngby, Denmark
[10] Laboratoire Central de la Peste, Institut Pasteur de Madagascar, BP1274, Antananarivo, Madagascar.

[†] Corresponding author: N.C. Stenseth: phone: +47-22854584/4400; fax: +47-22854001; e-mail: n.c.stenseth@bio.uio.no

Недавние события, связанные с Тяжелым Острым Респираторным Синдромом или атипичной пневмонией (SARS) [1] и птичьим гриппом показывают, что общественная и политическая реакция на угрозу, которую представляют новые антропозоонозы, может быть близкой к истерии. Это может привести к тому, что мы станем пренебрегать классическими зоонозными инфекционными болезнями, такими как чума (Рамка 1).

Непрекращающиеся проблемы, вызываемые чумой, стали темой трех недавних международных встреч. Мультидисциплинарная встреча по чуме, о ее настоящем, прошлом и будущем, была проведена в Академии Наук и Литературы в Осло, Норвегия (http://www.cees.no/oslo-plague-meeting). Основной темой симпозиума, организованного *Wellcome Trust Centre for the History of Medicine Трастовый Центр Истории Медицины* (Университетский Колледж, Лондон; http://www.ucl.ac.uk/histmed/) было сравнение «Черной Смерти» и современной чумы. Всемирная Организация Здравоохранения (ВОЗ) организовала встречу экспертов в Антананариву, Мадагаскар, для обсуждения и обновления клинических определений чумы, диагностических методов, вакцин и антибактериальной терапии, а так же природных очагов чумы и стратегии контроля ее переносчиков. (http://www.who.int/csr/disease/plague/interregionalmeeting2006/en/index.html).

Во время проведения этих встреч были сделаны несколько основных совместных заключений: (i) должно быть заново подчеркнуто, что возбудитель чумы (*Yersinia pestis*) все еще является причиной заболевания несколько тысяч человек в год [2, 3] (рис. 1); (ii) в местном масштабе восприятие рисков значительно превышает объективный риск, основанный на числе случаев [2]; (iii) изменение климата может увеличить вероятность вспышек чумы, там где в настоящее время имеются природные очаги и могут возникнуть новые ареалы чумы [2,4]; (iv) невероятно мало известно о динамике чумы в ее естественных носителях и следовательно и об изменении рисков для людей [5]; и, поэтому международное сообщество (v) должно относиться к чуме намного серьезнее, чем в настоящий момент.

Система чумы

Чумной микроб вызывает быстрое развитие тяжелого заболевания, которое в его бубонной форме без применения антибиотиков летально на 40-70 %; легочная форма и септицемия являются фактически всегда фатальными без лечения. По этим причинам *Y. pestis* считается одной из наиболее патогенных бактерий для людей. Возбудитель передается блохами, в то время как два других известных патогенных для людей вида *Yersinia* (*Y. enterocolitica* и *Y. псевдотуберкулеза*) передаются фекально-оральным путем и вызывают кишечные расстройства умеренной интенсивности. *Yersinia pestis*, как полагают, является клоном *Y. псевдотуберкулеза*, который появился в течение прошлых 1,500 – 20,000 лет [6, 7]. Это расхождение видов было характеризовано приобретением нескольких генетических элементов, а точнее, двух плазмид, которые играют ключевую роль при передаче заболевания блохами [8, 9]. Исключительную патогенность *Y. pestis* по сравнению с энтеропатогенными разновидностями можно объяснить ее новым способом передачи. Действительно, единственная возможность для этой бактерии перейти к новым хозяевам заключается в способности вызывать септицемию, позволяющую бактерии находиться в кровотоке и попасть в блоху во время ее кровавой трапезы [10].

Вскоре после открытия Иерсеном (Yersin) возбудителя чумы [11], стало ясно, что вспышки заболевания в поселениях были связаны с наличием инфекции у крыс и их блох. В этом классическом сценарии возникновения городской чумы инфицированные крысы (перевозимые, в частности, на судах) прибывают в новый город, и передают инфекцию местным домашним крысам и их блохам, которые, в свою очередь, служат источниками инфицирования человека. Иногда, у людей развивается легочная форма чумы, которая в дальнейшем передается от человека к человеку воздушно-капельным путем.

Эпидемиология чумы, однако, намного сложнее, чем предполагает данный сценарий городской чумы, и включает несколько других - более вероятных - путей передачи (Таблица 2). Это требует пересмотра экологии чумы.

Прошлое

Чума вызвала, по крайней мере, три основных пандемии. Первая (“чума Юстиниана”) распространилась в Средиземноморье в 6-ом столетии нашей эры, вторая (“Черная Смерть”) началась в Европе в 14-ом столетии и возвращалась периодически в течение более 300 лет, и третья началась в Китае в середине 19-ого столетия и распространилась по всему миру. Предполагают, что пандемии были вызваны различными биоварами *Yersinia pestis*, соответственно Antiqua (до сир пор существующего в Африке и Центральной Азии), Medievalis (в настоящее время распространенного в Центральной Азией) и Orientalis (почти во всем мире) [12, 13].

Черная Смерть опустошала Средневековую Европу, и как результат оказала значительное влияние на социально-экономическое развитие континента, культуру, искусство, религию и политику [14, 15]. В нескольких критических анализах подвергли сомнению, была ли Черная Смерть действительно вызвана *Y. pestis* [16, 17]. И в Осло, и на Лондонских встречах встретились сторонники обоих мнений. Они пришли к согласию, что эпидемиология Черной Смерти, как отражено в исторических отчетах, не всегда соответствовала «классическому» циклу чумы, крыса-блоха-человек. Однако, симптомы заболевания, о которых имеется информация, были очень похожими при каждой пандемии, и международный опыт современной чумы, представленный на встрече в Осло показал, что «классическая» эпидемиология чумы - только одна из нескольких возможностей, и может быть она не является типичной [18]. Обнаружение генетического материала *Y. pestis* жертв Черной Смерти, похороненных в средневековых могилах [19], подтверждает мнение о том, что *Y. pestis* была возбудителем Черной Смерти.

Настоящее

Историческая важность чумы часто приводила к тому, что чума классифицировалась, как проблема прошлого. Однако, она остается существующей угрозой во многих частях мира (рис. 1a), особенно в Африке, где и число случаев возникновения болезни (Рис. 1b), и число стран в которых она регистрируется (Рис. 1c) увеличилось в течение последних десятилетий. После нового появления чумы в 90-х годах в нескольких странах, чума была характеризована, как вновь возникающая болезнь [20, 21].

Разные виды диких грызунов во всем мире, живущие в широком диапазоне естественных сред обитания, являются носителями чумы, в то время как городские крысы только иногда играют роль «связных», перенося чуму между дикой природой и людьми. Были зарегистрированы и другие пути передачи от животного к человеку. Человеку чума может передаваться (i) от блох диких грызунов в сельских районах (например, в юго-западных Соединенных Штатах [22, 23]) или блох грызунов коменсалов, которые свободно передвигаются между деревнями и лесной средой обитания, в которых обитают носители (например, Танзания): передвижения, становятся более частыми, так как человеческая деятельность привела к фрагментному уничтожению леса [24]; (ii) через употребление в пищу инфицированных животных, таких как морские свинки в Перу и Эквадоре [25, 26] или верблюдов, которые заражаются от блох грызунов в Центральной Азии и на Ближнем Востоке [27-31]; или (iii) от контакта с кошками, инфицированными при поедании зараженных чумой грызунов в Африке или Соединенных Штатах [32-34]. Передача от человека к человеку тоже происходит, или непосредственно воздушно-капельным путем или косвенно, через укусы блохи [35-37].

За последние 20 лет ВОЗ регистрирует от 1000 до 5000 человеческих случаев заболевания и 100 - 200 смертных случаев в год [38]. Однако, из-за низкого уровня диагностики и занижения сведений, число случаев наверняка намного выше. За эти годы, основное количество заболеваний переместилось из Азии в Африку (Рис. 1b), более 90 % всех случаев заболеваний и смертей за последние пять лет произошли в Мадагаскаре, Танзании, Мозамбике, Малави, Уганде и Демократической Республике Конго. В большинстве это случаи бубонной формы чумы, вызванные контактом с зараженными грызунами и блохами. Однако все еще происходят вспышки легочной чумы, самая последняя произошла в мае-июле 2006 года в ДРК с более чем 200 предполагаемыми случаями (http://www.who.int/csr/don/2006_06_14/). В декабре 2004 г. была вспышка легочной чумы в лагере шахтеров в ДРК, вероятно завезенная инфицированным человеком, который приехал из эндемичного района. Один заболевший легочной формой чумы даже появился в Кисангани, это большой город на расстоянии в несколько сотен километров от первоначального места [39, 40]. Даже бригады врачей быстрого реагирования не могут остановить распространение чумы на большие расстояния прежде, чем она будет обнаружена и взята под контроль.

Африка особенно опасна по ряду причин. Бедные сельские общины обычно живут в непосредственной близости к грызунам. На грызунов охотятся и их употребляют в пищу во многих эндемичных по чуме областях. Суеверия, недостаток денег и большие расстояния от медицинских учреждений часто приводят к запоздалому обращению за медицинской помощью. Система здравоохранения в большей части Африки плохо организована и оборудована, а политический кризис и социальная дезорганизация препятствуют ее усовершенствованию. И, наконец, антропогенные изменения в ландшафте и в способах передвижений людей все более и более провоцируют контакты между носителями чумы и полу-домашними грызунами, и между людьми из эндемичных по чуме районов и людными проживающими в ранее свободных от чумы регионах.

Будущее

Чума не может быть уничтожена, так как она широко распространена в популяциях диких грызунов. Следовательно, необходимо осознать каким образом динамика развития этих районов живой природы влияет на риски для человека. Например, вероятность вспышки чумы среди популяции грызунов в Северной Америке и Центральной Азии, представляющей опасность для населения, как известно, связана с климатическими изменениями [41, 42]. Недавний анализ данных из Казахстана [43] показывает, что более теплые весны и более влажное лето увеличивают распространенность чумы в популяции ее главного хозяина, большой песчанки. Такие экологические условия, кажется, преобладали в течение второй и третьей чумной пандемии [44] - условия, которые могли бы стать более типичными в будущем [45].

Хотя число человеческих случаев чумы относительно низко, было бы ошибочно недооценить ее угрозу для человечества, из-за ее высокой контагиозности, быстрого распространения, быстрого клинического течения и высокой смертности при отсутствии лечения. Вспышка чумы может также привести к возникновению паники, как произошло в Индии в 1994 в Сурате, при относительно небольшой вспышке с 50 смертными случаями [46]. Это привело к упадку в туристической сфере и торговле по всей стране, и ущербу в 600 миллионов долларов [47].

Работа во время вспышки обычно сходна с борьбой с пожаром. Команды прибывают в зараженную местность для уничтожения блох инсектицидами, для оказания помощи больным и для выполнения профилактических процедур. Многие эксперты утверждали, что такой подход кризисного управления недостаточен, поскольку пока будут предприняты какие-либо действия вспышка, вероятно, уже будет убывать. Информирующие и упреждающие решения о профилактических мероприятиях до возникновения заболеваний, конечно, были бы более действенными и экономически выгодными методами решения проблемы. В последнее время был достигнут некоторый прогресс в области разработки быстрых методов диагностики [48], некоторые из которых ставят под сомнения общепринятые догмы о динамике развития чумы в дикой природе Соединенных Штатов [49] и Центральной Азии [50], и идентификацию прогнозируемых критических порогов популяций грызунов для чумы в Казахстане [51]. Поразительно то, что мы недостаточно понимаем эту сложную болезнь даже в хорошо изученных очагах, в частности в Африке: часто, мы даже не знаем ее природного источника.

Способность возбудителя чумы формировать постоянные очаги при очень разнообразных экологических условиях говорит о ее необычной адаптируемости. В течение ее появления в Центральной Азии, *Y. pestis* приобрела множественные копии инсерционных сегментов, которые приводили ее геном в состояние чрезвычайной гибкости [52]. Способность подвергаться генным перестановкам может являться эффективным средством, позволяющим возбудителю чумы приспособиться к новым экологическим нишам. Кроме того, как было недавно обнаружено, *Yersinia pestis* способна приобретать плазмиды резистентности к антибиотикам в естественных условиях [53, 54], вероятно во время прохождения средней кишке блохи [55]. Очевидно, что появление и распространение штаммов *Y. pestis* с множественной устойчивостью представило бы главную угрозу человеческому здоровью.

Наконец, мы не должны недооценивать тот факт, что чума была в прошлом использована как биооружие начиная от катапультирования трупов через городские стены, до сбрасывания инфицированных блох с самолетов и до современных аэрозольных установок [56, 57]. Разработки по созданию боевой чумы, проходившие со времен Второй Мировой Войны до 1990-х годов, вызвали страх перед возможностью использования биооружия, что в свою очередь стимулировало исследования в области разработки стратегии надзора и реагирования. Позже, однако, опасения относительно актов биотерроризма более мелкого масштаба, а также желание государственных органов более полно контролировать любой доступ к материалу, привело к опасности ограничения исследований в области экологии и эпидемиологии чумы, которые

необходимы для улучшения клинической работы в эндемичных районах. Распыление аэрозоля в террористических целях в ограниченном помещении может вызвать значительное количество смертельных случаев и привести к широкомасштабной панике [56, 57], и никто не хочет, чтобы чума была использована, как биооружие и чтобы она попала в руки террористов. Однако, сейчас, как никогда существует потребность в проведении научных исследований по изучению динамики трансмиссии, контроля вспышек и улучшении надзора за этой инфекцией.
Может чума не сравнима с массовыми болезнями-убийцами (малярия, СПИД ВИЧ, ТБ; см., например, [58]) по числу текущих случаев заболевания, но она гораздо более патогенна и способна быстро распространиться при благоприятных условиях. Легко забыть чуму в 21-ом столетии, воспринимая ее, как историческое событие. Но по нашему мнению, чума не должна отодвигаться на второй план. Она остается недостаточно изученной угрозой, которую мы не имеем право игнорировать.

Библиография

1. McLean, A.R., May, R.M., Pattison, J. & Weiss, R.A. (2005) *SARS. A case study in emerging infections*. Oxford University Press, Oxford.
2. World Health Organisation (2003) Plague. *Weekly Epidemiological Record*, **78**, 253-260.
3. World Health Organisation (2005) Plague. *Weekly Epidemiological Record*, **80**, 138-140.
4. Stapp, P., Antolin, M.F. & Ball, M. (2004) Patterns of extinction in prairie dog metapopulations: plague outbreaks follow El Nino events. *Frontiers in Ecology and the Environment* **2**, 235–240.
5. Gage, K.L. & Kosoy, M.Y. (2005) Natural history of plague: perspectives from more than a century of research. *Annual Review of Entomology* **50**, 505–528.
6. Achtman, M., Zurth, K., Morelli, G., Torrea, G., Guiyoule, A. & Carniel, E. (1999) *Yersinia pestis*, the cause of plague, is a recently emerged clone of *Yersinia pseudotuberculosis*. *Proceedings of National Academy of Science, Washington* **96**, 14043-14048.
7. Achtman, M., Morelli, G., Zhu, P., Wirth, T., Diehl, I., Kusecek, B., Vogler, A.J., Wagner, D.M., Allender, C.J., Easterday, W.R., Chenal-Francisque, V., Worsham, P., Thomson, N.R., Parkhill, J., Lindler, L.E., Carniel, E., & Keim, P. (2004) Microevolution and history of the plague bacillus, *Yersinia pestis*. *Proceedings of National Academy of Science, Washington* **101**, 17837–17842.
8. Sodeinde, O. A. et al. (1992) A surface protease and the invasive character of plague. *Science* **258**, 1004-1007.
9. Hinnebusch, B. J. *et al.* (2002) Role of *Yersinia* murine toxin in survival of *Y. pestis* in the midgut of the flea vector. *Science* **296**, 733-735.
10. Carniel, E. (2003) Evolution of pathogenic Yersinia, some lights in the dark. *Advances in Experimental Medical Biology* **529**, 3-12.
11. Yersin, A. (1894) La peste bubonique à Hong-Kong. *Ann. Inst. Pasteur* **2**, 428-430.
12. Devignat, R. (1951) Variétés de l'espèce *Pasteurella pestis*. Nouvelle hypothèse. *Bulletin de l'Organisation Mondiale de la Santé* **4**, 247-263.
13. Guiyoule, A. Grimont, F., Iteman, I., Grimont, P.A.D., Lefevre, M. & Carniel, E. (1994) Plague pandemics investigated by ribotyping of *Yersinia pestis* strains. *Journal of Clinical Microbiology* **32**, 634-641.
14. Twigg, G. (1984) *The Black Death: a biological reappraisal*. Batsform Academic and Educational, London.
15. Ziegler, P. (1969) *The Black Death*. Alan Sutton Publishing Inc., Wolfeboro Falls, NH.
16. Scott, S. & Duncan, C.J. (2001) *Biology of Plagues: Evidence from Historical Populations.* Cambridge University Press, Cambridge.
17. Cohn, Jr., Samuel K. (2002) *The Black Death Transformed*. Arnold, London.

18. Drancourt, M. (2006) *Yersinia pestis* as a telluric, human ectoparasite-borne organism. *Lancet Infectious Disease* **6**, 234-241.
19. Raoult, D., Aboudharam, G., Crubézy, E., Larrouy, G., Ludes, B. & Drancourt, M. (2000) Molecular identification by "suicide PCR" of *Yersinia pestis* as the agent of Medieval Black Death. *Proceedings of National Academy of Science, Washington* **97**, 12800–12803.
20. Schrag, S. J. & Wiener, P. (1995) Emerging infectious diseases: what are the relative roles of ecology and evolution? *Trends in Ecology and Evolution* **10**, 319-324.
21. Duplantier, J. M., Duchemin, J. B., Chanteau, S. & Carniel, E. (2005) From the recent lessons of the Malagasy foci towards a global understanding of the factors involved in plague reemergence. *Veterinary Research* **36**, 437-453.
22. Gage, K.L., Ostfeld, R.S. & Olson, J.G. (1995) Nonviral vector-borne zoonoses associate with mammals in the United States. *Journal of Mammalogy* **76**, 695-715.
23. Levy, C.E, & Gage, K.L (1999). Plague in the United States 1995-1996. *Infections in Medicine* **16**, 54-64.
24. Kaoneka, A.R.S & Solberg, B. (1994) Forestry-related land use in the West Usambara mountains, Tanzania. *Agriculture, Ecosystems and Environment*. **49**, 207-215.
25. Gabastou J-M, Proano J, Vimos A, Jaramillo G, Hayes E, Gage K, Chu M, Guarner J, Zaki S, Bowers J, Guillemard C, Tamayo H, and Ruiz A. An outbreak of plague including cases with pneumonic infection, Ecuador, 1998. *Transactions of the Royal Society of Tropical Medicine and Hygiene* **94**, 387-391 (2000).
26. Ruiz A. (2001) Plague in the Americas. *Emerging Infectious Diseases* **7**, (Supplement) 539-540.
27. Fedorov V.N. (1960) Plagues in camels and its prevention in the USSR *Bulletin of WHO* **23**, 275-281.
28. Christie, A.B., Chen, T.H.& Elberg, S.S.(1980) Plague in camels and goats: their role in human epidemics. *Journal of Infectious Disease* **141**, 724-726.
29. Atshabar B. B., Aikimbaev A.M., Aubakirov S.A., Suleimenov B.M. et al. (2000) Epizootologic and social basis for plague human infection in 1999 in Kazakhstan and their clinical-epidemiologic characteristics. *Problems of the most dangerous infections*, Saratov, 14-21.
30. Arbaji, A, Kharabsheh, S., Al Azab, S., Al Kayed, M., Amr, Z.S., Baker, M.A. & Chu, M.C. (2005) A 12-case outbreak of pharyngeal plague following the consumption of camel meat, in north-eastern Jordan. *Annals of Tropical Medicine and Parasitology* **99**, 789-793.
31. Bin Saeed, A.A., Al-Hamdan, N.A. (2005) Fontaine RE. Plague from eating raw camel liver. *Emerging Infect Disease* **11**, 1456-1457.
32. Isaacson, M. (1973) Unusual cases of plague in southern Africa. *South African Medical Journal* **47**, 2109-2113.
33. Doll, J. M., Seitz, P. S., Ettestad, P., Bucholtz, A. L., Davis, T., & Gage, K.L. (1994) Cat Transmitted Fatal Pneumonic Plague in a Person Who Travelled from Colorado to Arizona. *American Journal of Tropical Medicine and Hygiene* **51**, 109-114.
34. Gage, K.L., Dennis, D.T., Orloski, K.A., Ettestad, P.J., Brown, T.L., Reynolds, P.J., Pape, W.J., Fritz, C.L., Carter, L.G. & Stein, J.D. (2000) Cases of cat-associated human plague in the Western US, 1977-1998. *Clinical Infectious Diseases* **30**, 893-900.
35. Pollitzer R. (1954) Plague. *World Health Organization Monograph Series* **22**, 1-698.
36. Pollitzer, R. (1960) A review of recent literature on plague. *Bulletin of WHO* **23**, 313-400.
37. Blanc G. (1956) Une opinion non conformiste sur le mode de transmission de la peste. *Revue d'Hygiéne et de Médecine Sociale* **4**, 532-562.
38. World Health Organisation (2004) Human Plague in 2002 and 2003. *Weekly Epidemiological Record* **79(33)**, 301-306.

39. Bertherat, E., Lamine, K.M., Formenty, P., Thullier, P., Mondonge, V., Mitifu, A. & Rahalison, L. (2005) Epidémie de peste pulmonaire dans un camp minier de la République Démocratique du Congo: le réveil brutal d'un vieux fléau. *Médecine Tropicale* **65**, 511-514.
40. Parmenter, R.R., Yadav, E.P., Parmenter, C.A., Ettestad, P.J. & Gage, K.L. (1999) Incidence of plague associated with increased winter-spring precipitation in Mexico. *American Journal of Tropical Medicine and Hygiene* **61**, 814-821.
41. Enscore, R.E., Biggerstaff, B.J., Brown, T.L., Fulgham, R.F., Reynolds, P.J., Engelthaler, D.M., Levy, C.E., Parmenter, R.R., Montenieri, J.A., Cheek, J.E., Grinnell, R.K., Ettestad, P.J. & Gage, K.L. (2002) Modeling relationships between climate and the frequency of human plague cases in the southwestern United States, 1960–1997. *American Journal of Tropical Medicine and Hygiene* **66**, 186–196.
42. Stenseth, N.C., Samia, N.I., Viljugrein, H., Kausrud, K., Begon, M. Davis, S., Leirs, H., Dubyanskiy, V.M, Esper, J., Ageyev, V.S., Klassovskiy, N.L., Pole, S.B. & Chan, K.-C. (2006) Plague Dynamics are driven by Climate Variation. *Proceedings of National Academy of Science, Washington* **103**, 13110-13115.
43. Treydte, K., Schleser, G.H., Helle, G., Frank, D.L., Winiger, M., Haug, G.H. & Esper, J. (2006) Twentieth century as the wettest period in northern Pakistan over the past century. *Nature* **440**, 1179-1182.
44. IPCC. *Climate Change 2001: The Scientific Basis. Contribution of Working Group I to the Third Assessment Report of the Intergovernmental Panel on Climate Change* [Houghton, J.T., Ding, Y, Griggs, D.J., Noguer, M., van der Linden, P.J., Dai, X., Maskell, K. & Johnson, C.A. (eds.)]. Cambridge University Press, Cambridge, United Kingdom and New York, NY, USA, 881pp.
45. Ganapati, M. (1995) India's pneumonic plague outbreak continues to baffle. *British Medical Journal* **311**, 706.
46. Fritz, C.L., Dennis, D.T., Tipple, M.A., Campbell, G.L., McCance, C.R., & Gubler, D.J. (1996) Surveillance for Pneumonic Plague in the United States During an International Emergency: A Model for Control of Imported Emerging Diseases. *Emerging Infectious Diseases* **2**, 30-36.
47. Chanteau S., Rahalison L., Ralafiarisoa L., Foulon F., Ratsitorahina M., Ratsifasoamanana L., Carnie E. & Nato F. (2003) Development and testing of a rapid diagnostic test for bubonic and pneumonic plague. *The Lancet,* **361**, 211-216.
48. Webb, C.T., Brooks, C.P., Gage, K.L. & Antolin, M.V. (2006) Classic flea-born transmission does not drive plague epizootics in prairie dogs. *Proceedings of National Academy of Science*, *Washington* **103**, 6236-6241.
49. Begon, M., Klassovskiy, N., Ageyev, V., Suleimenov, B., Atshabar, B. & Bennett, M. (2006) Epizootiologic parameters for plague in Kazakhstan. *Emerging Infectious Diseases* **12**, 268-273.
50. Davis, S., Begon, M., De Bruyn, L., Ageyev, V., Viljugrein, H., Stenseth, N. & Leirs, H. (2004) Predictive Thresholds for Plague in Kazakhstan. *Science* **304**, 736–738.
51. Parkhill, J., Wren, B.W., Thomson, N.R. *et al.* (2001) Genome sequence of *Yersinia pestis*, the causative agent of plague. *Nature* **413**, 523-527.
52. Galimand, M., Guiyoule, A., Gerbaud, G., Rasoamanana, B., Chanteau, S., Carniel, E. & Courvalin, P. (1997) Multidrug resistance in *Yersinia pestis* mediated by a transferable plasmid. *New England. Journal of Medicine* **337**, 677-680.
53. Guiyoule, A. Gerbaud, G., Buchrieser, C., Galimand, M., Rahalison, L., Chanteau, S., Courvalin, P. & Carniel, E. (2001) Transferable plasmid-mediated resistance to streptomycin in a clinical isolate of *Yersinia pestis*. *Emerging Infectious Disease* **7**, 43-48.

54. Hinnebusch, B. J., Rosso, M. L., Schwan, T. G. & Carniel, E. (2002) High-frequency conjugative transfer of antibiotic resistance genes to *Yersinia pestis* in the flea midgut. *Molecular Microbiology* **46**, 349-354.
55. Inglesby, T.V., Dennis, D.T., Henderson, D.A., Bartlett, J.G., Ascher, M.S., Eitzen, E., Fine, A.D., Friedlander, A.M., Hauer, J., Koerner, J.F., Layton, M., McDade, J., Osterholm, M.T., O'Toole, T. Parker, G., Perl, T.M. Russell, P.K., Schoch-Spana, M., Tonat, K. (2000) Plague as a Biological Weapon: Medical and Public Health Management. *Journal of the American Medical Association* **283**, 2281-2290.
56. Koirala, J. (2006) Plague: disease, management, and recognition of act of terrorism. *Infectious disease clinics of North America* **20**, 273-287.
57. Hotez, P.J., Molyneux, D.H., Fenwick, A., Ottesen, E., Sachs, S.E. & Sachs, J.D. (2006) Incorporating a reapid-impact package for neglected tropical diseases with programs for HIV/AIDS, Tuberculosis, and Malaria. *PloS Medicine* **3**, 576-584.
58. Simond, P.L. (1898) La propagation de la peste. *Annales de l'Institut Pasteur* **12**, 625-687.
59. Baltazard, M., Karimi, Y., Eftekhari, M., Chamsa, M. & Mollaret, H. H. (1963) La conservation interépizootique de la peste en foyer invétéré hypothèses de travail. *Bulletin de la Société de Pathologie Exotique* **56**, 1230-1241.

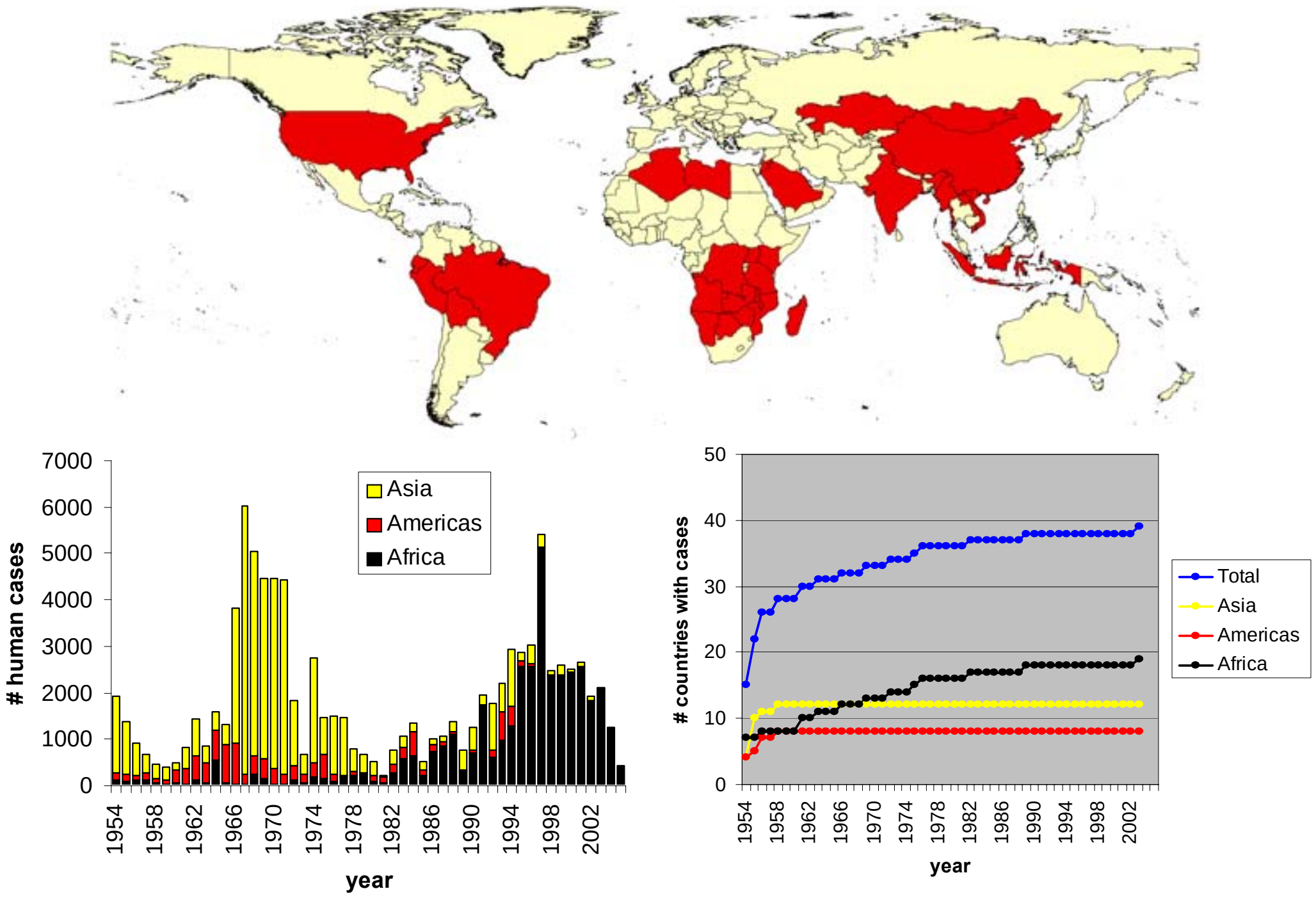

Рисунок 1: Распределение чумы в мире. (a) На карте указаны страны, которые сообщают о чуме с 1975 года (оранжевый) и области с известным природными очагами чумы (красный) ([3]) (для США показанна только территория южнее 50 градусов северной долготы); (b) Ежегодное число случаев чумы у людей по различным континентам, по сообщениям ВОЗ в период 1954-2005; (c) Общее число стран, которые сообщили о чуме в ВОЗ с 1954.

Рамка 1: Чума

(231 слово)

Бактерия, вызывающая заболевание чумой (*Yersinia pestis*), была обнаружена Иерсеном (Yersin) в 1894 году [11] (см. также [58]). Соотношение заболевание-смертность варьируется от 30 до 100 %, при отсутствии лечения. Чума эндемична для многих стран в Америке, Азии и Африке, в настоящее время более чем 90 % случаев заболеваний приходится на Африку.

Клиническое описание: После инкубационного периода в 3-7 дней, пациенты обычно испытывают внезапное начало лихорадки, озноб, головные боли, боли в теле, слабость, рвоту и тошноту. В зависимости от путей инфицирования и клинической картины различают 3 клинические формы чумы: бубонная, септическая и легочная. Бубонная форма - самая распространенная форма, возникающая вследствие укуса инфицированной блохи. Легочная - самая опасная и наименее распространенная, обычно вызванная вторичным распространением бубонной инфекции. Первичная легочная чума может передаваться напрямую от человека-человеку воздушно-капельным путем.

Лечение: Быстрый диагноз и лечение очень важны для уменьшения осложнений и смертности. Стрептомицин, тетрациклины и сульфаниламиды остаются главными препаратами лечения бубонной формы чумы. Гентамицин и фторхинолоны применяются как альтернативные препараты, когда вышеупомянутые антибиотики недоступны. Пациенты с легочной формой чумы должны изолироваться с целью предотвращения респираторной передачи болезни.

Недавние достижения Диагностика чумы остается проблемой, потому что большинство случаев заболевания людей происходит в отдаленных районах с недостаточными лабораторными ресурсами. Пока, главные методы подтверждения диагноза были основаны на выделении *Y. pestis* (требующими, как минимум, 4 дня). Недавние разработки быстрых диагностических препаратов, планируемых к использованию, как подтверждающих методов в эндемичных районах, открывают новые перспективы в области надзора и реагирования.

Рамка 2: Бактерия чумы в пределах экологической системы
(164 слова)

Сохранение очагов чумы зависит от всей группы грызунов и связанных с ними блох (Рис. 2 (цикл a)). При благоприятных условиях, возбудитель чумы может выжить в окружающей среде в норах грызунов [59]. Когда инфицированная блоха питается кровью домашнего грызуна, возбудитель продолжает в нем цикл развития (Рис. 2 (цикл b)). После смерти домашнего грызуна, блохи вынуждены перемещаться на альтернативных хозяев, например людей. Если у людей развивается легочная форма чумы, инфекция может передаваться от человека к человеку воздушно-капельным путем (Рис. 2 (цикл c)). Люди также могут заразиться при контакте с инфицированными животными (или через мясо), включая грызунов, кроликов, верблюдов, или кошек. У кошек также может развиться легочная чума, передающаяся их владельцам через кашель. И, наконец, есть основания предполагать, что человеческая блоха *Pulex irritans* может участвовать в передаче инфекции от человека-человеку [37]. Млекопитающие хищники, хищные и другие птицы, использующие норы грызунов для своих гнезд, могут передвигаться на гораздо большие расстояния, чем сами грызуны, распространяя инфекцию на обширные территории. Также, инфицированные крысы-комменсалы и люди могут перемещаться на большие расстояния.

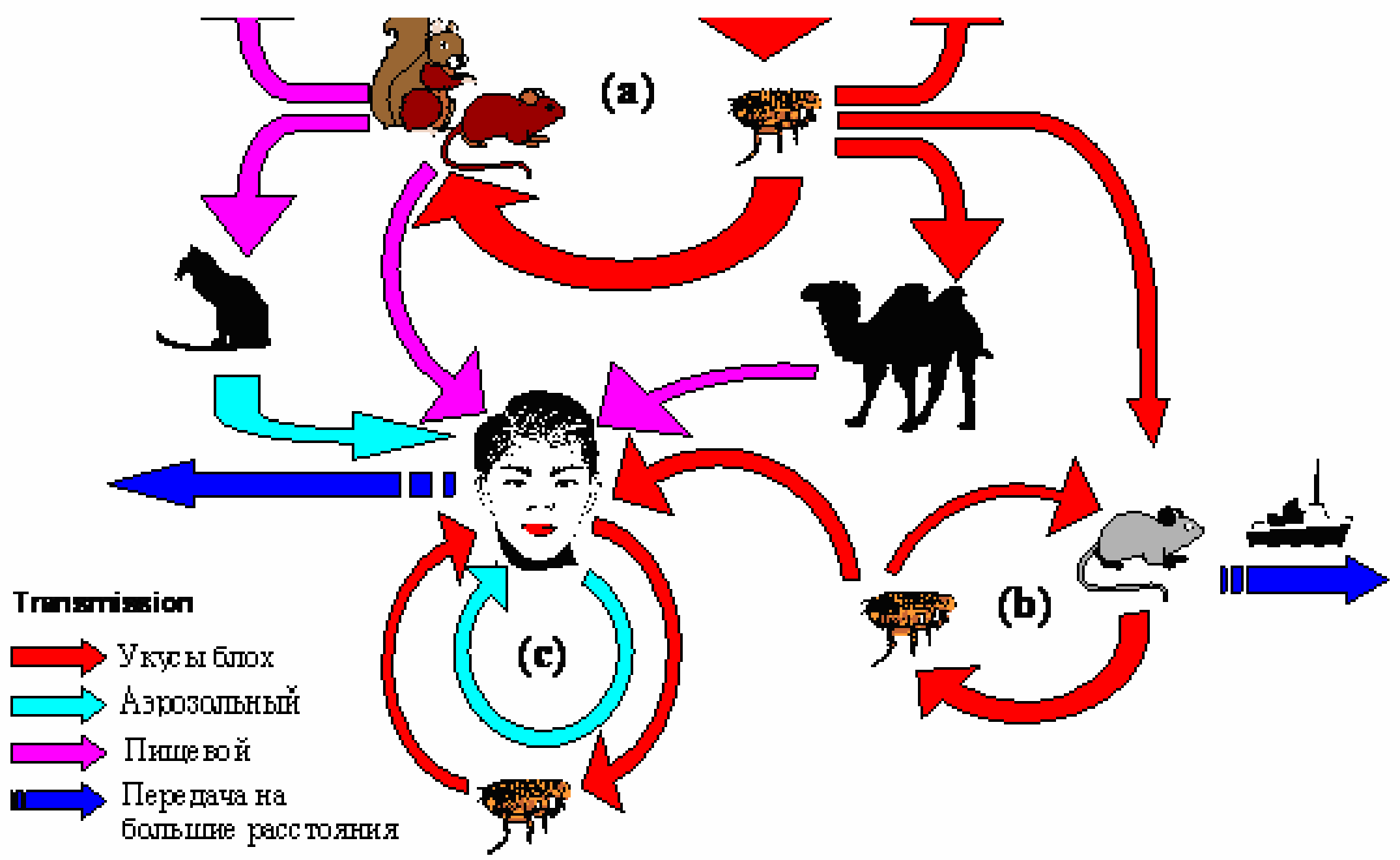

Рисунок 2: Возможные пути передачи возбудителя чумы *Y. pestis*, включая цикл в дикой природе: грызун - блоха (a), цикл с участием грызунов-комменсалов: блоха - грызун (b) и передача легочной формы среди людей (c). Цвет стрелок указывает на механизм передачи (укусы блох, аэрозольный, пищевой (потребление мяса) бактерий от одного хозяина к другому. Темные синие стрелки указывают пути передвижения чума в другие районы.
